# Research Progress in the Multilayer Hydrogels

**DOI:** 10.3390/gels7040172

**Published:** 2021-10-17

**Authors:** Lu Jin, Jia Xu, Youcai Xue, Xinjiang Zhang, Mengna Feng, Chengshuang Wang, Wei Yao, Jinshan Wang, Meng He

**Affiliations:** School of Materials Science and Engineering, Yancheng Institute of Technology, Yancheng 224051, China; jinlu525@126.com (L.J.); xujia202109@163.com (J.X.); xyc1246@163.com (Y.X.); zhangxinjiang1983@163.com (X.Z.); fengmengnafwch@126.com (M.F.); wangcs@ycit.cn (C.W.); xiaoniu1981@126.com (W.Y.); wangjinshan@ycit.cn (J.W.)

**Keywords:** multilayer hydrogels, composites, fabrication process, mechanisms, application

## Abstract

Hydrogels have been widely used in many fields including biomedicine and water treatment. Significant achievements have been made in these fields due to the extraordinary properties of hydrogels, such as facile processability and tissue similarity. However, based on the in-depth study of the microstructures of hydrogels, as a result of the enhancement of biomedical requirements in drug delivery, cell encapsulation, cartilage regeneration, and other aspects, it is challenge for conventional homogeneous hydrogels to simultaneously meet different needs. Fortunately, heterogeneous multilayer hydrogels have emerged and become an important branch of hydrogels research. In this review, their main preparation processes and mechanisms as well as their composites from different resources and methods, are introduced. Moreover, the more recent achievements and potential applications are also highlighted, and their future development prospects are clarified and briefly discussed.

## 1. Introduction

Hydrogel is a kind of hydrophilic material with a three-dimensional crosslinked network structure, which is infiltrated with water [1]. It can absorb water quickly and retain water for a certain period without dissolving in water. The properties of hydrogels are similar to those of biological tissues and their excellent biocompatibility makes them extremely suitable for biomedical research [2,3,4]. According to different formation mechanisms and molecular structures, hydrogels can be generally divided into chemically crosslinked hydrogels and physically crosslinked hydrogels [5]. Chemically crosslinked hydrogels are formed through crosslinking with chemical bonds, which is an irreversible permanent crosslinking, and show extraordinary chemical stability with good properties such as good solvent resistance. Physically crosslinked hydrogels are noncovalently crosslinked and usually exhibit excellent properties such as reversibility, repairability, and high responsiveness to external stimuli, which can be modified further to endow them with other properties as “intelligent” materials [6,7]. It has been noted that hydrogels are widely used in different fields of drug delivery, tissue engineering, medical implants, wound dressings, and various mechanical and electronic devices due to their extreme mechanical properties, such as high degrees of toughness, robustness, elasticity, stickiness, and fatigue resistance [8,9,10,11,12,13,14].

The research and design of polymer-based hydrogels are mainly based on the overall consideration of their properties, and the resultant hydrogels are usually homogeneous materials [15]. However, it is challenging for these homogeneous hydrogels to simultaneously meet the needs of further microstructure control and different applications, including precise drug delivery and release of different drugs, bone repair and regeneration, and carriers of different cells. As a kind of heterogeneous hydrogel, multilayer hydrogels have emerged and become an important and novel branch of hydrogels. Multilayer hydrogels exhibit many excellent properties such as high ductility, unique complex internal structure, and excellent response to stimuli [16,17,18]. Moreover, multilayer hydrogels exhibit a variety of different shapes, such as spherical, cylindrical, spindle-like, and multilayer tubular, to satisfy different applications [19,20,21,22]. The internal structure of multilayer hydrogels is complex, which could exhibit cavities between each layer (namely inter-layer space) for the storage of drugs, microorganisms, and cells. Different layers can be prepared from different substances or methods to independently exhibit different physical and chemical properties [23].

In this review, we summarize the recent progress of the multilayer hydrogels and their composites. Particularly, we review the preparation processes and mechanisms of multilayer hydrogels and their innovative applications in different fields, especially in the biomedical field, including drug delivery, cell carrier or encapsulation, wound dressings (coatings), and bone repair. Finally, we provide a brief perspective on the future development of multilayer hydrogels, hoping to provide some theoretical guidance on broadening hydrogels’ application.

## 2. Preparation Methods and Mechanisms of the Multilayer Hydrogels

Many different preparation methods for multilayer hydrogels have been developed in recent years according to different raw materials, crosslinking structures (chemical or physical), and potential applications. These methods can be generally divided into two pathways according to the formation direction of hydrogel layers: from the inside to the outside and from the outside to the inside.

More recently, the preparation of multilayer hydrogels from inside to outside has been widely studied, and its main preparation technology is as follows: First, suitable materials are selected to prepare inner gel cores (generally agarose) with special shapes according to application requirements. Then, a first gel layer is formed on the gel core by different crosslinking or coagulation methods through pretreating the gel core with subsequent soaking in certain solutions. Finally, the fabrication process is repeated several times to generate multilayer hydrogels with the desired number of layers. The volume of multilayer hydrogels continues to grow until the preparation process of the multilayer structure is completed by gradually wrapping or covering the gel cores with gel layers.

Compared with the pathway from inside to outside, the preparation of multilayer hydrogels from outside to inside has been studied much less, although this form appeared earlier. The main preparation process is as follows: First, suitable raw materials are selected to prepare the hydrogel shells, which can wrap the solutions corresponding to the following gel layers. Second, a first gel layer is formed on the inner wall of the shell by different coagulation or crosslinking methods. Finally, the above fabrication process is repeated several times to generate multilayer hydrogels with the desired number of layers. The total volume of multilayer hydrogels prepared from outside to inside cannot increase after the formation of the shells. All the gelation processes occur inside the shells and move from the shell to the center of the gel with a gradual layer-by-layer (LBL) formation.

Detailed preparation methods are commonly used for the above two pathways, which can be roughly divided into the lLBL method and non-LBL method, as follows.

### 2.1. LBL Assembling Methods for Multilayer Hydrogels

At present, LBL assembly is the most widely used method for the preparation of multilayer hydrogels from different polymers such as polysaccharides. LBL assembly can be used to prepare multilayer structures by different driving forces or crosslinking types. Different LBL assembly methods are suitable for the preparation of multilayer hydrogels with different performances and application requirements. In the following discussion, several commonly used LBL methods are analyzed from the aspects of preparation principle, advantages, and application prospects.

#### 2.1.1. LBL by Chemical Crosslinking

**Figure 1:**
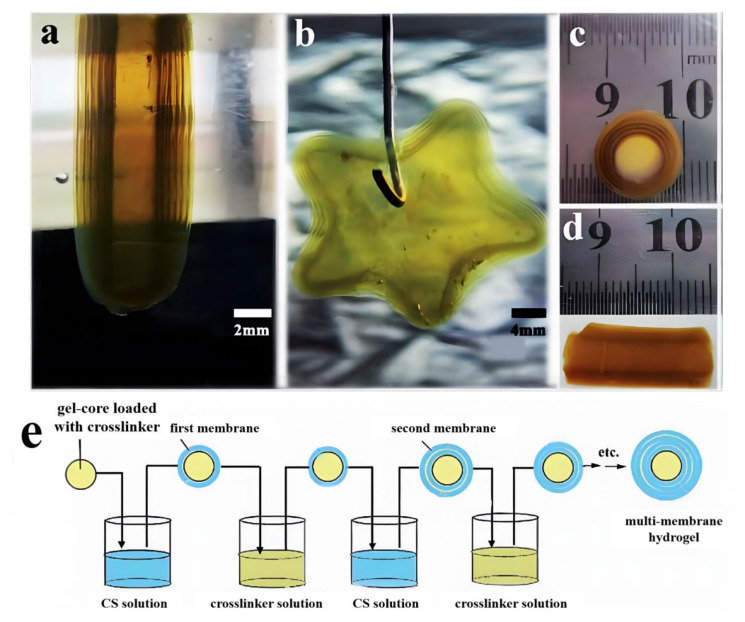
Chitosan multilayer hydrogels with various shapes: column (**a**), star (**b**), and tubular (**c**,**d**), and scheme of the preparation process of chitosan multilayer (multi-membrane) hydrogels by the LBL assembly method (**e**). (Reproduced with permission from [24]. Royal Society of Chemistry, 2013).

Chemical crosslinking is a common method of preparing homogenous hydrogels via the use of crosslinking agents. Interestingly, multilayer hydrogels with arbitrary shapes, including onion-like, tubular, and star-like, can be readily prepared using the chemical crosslinking method (Figure 1a–d) through the LBL process. As an example, the preparation process of a chitosan multilayer hydrogel from Xiong’s group is roughly shown in Figure 1e [24]. First, suitable raw materials (e.g., agarose) were selected to prepare a gel core, which was immersed in the crosslinker solutions (such as glutaraldehyde, terephthalaldehyde, and epoxy chloropropane) for an appropriate time to load the crosslinker. Second, the agarose gel core loaded with the crosslinker was soaked in the chitosan solution for a required time to crosslink the neighboring chitosan chains for the formation of the first chitosan gel layer. Third, the obtained gel core with the first chitosan gel layer was immersed in the crosslinker solution again for the desired time to ripen the chitosan gel layer and load the crosslinker for the formation of the second chitosan gel layer, followed by soaking in the chitosan solution. Onion-like CS multilayer (multi-membrane) hydrogels with the desired layers can be prepared by repeating the above-mentioned process. The chitosan layers were covalently crosslinked with chemical crosslinkers to endow them with good solvent resistance and pH sensitivity.

For chemically crosslinked multilayer hydrogels by the LBL process, reasonable and rapid crosslinking is essential for successful preparation. The formation and growth of each gel layer are related to the diffusion of the crosslinker. The inter-layer space canbe adjusted by changing the crosslinking degree of gel layers. Moreover, the chemically cross-linked chitosan multilayer hydrogels have a unique sub-layer structure [24]. The chitosan multilayer hydrogels have pH sensitivity and can disintegrate layer by layer, thus showing promise for applications in different fields, including drug delivery and tissue engineering, due to their unique structure.

#### 2.1.2. LBL by Ion Crosslinking

**Figure 2:**
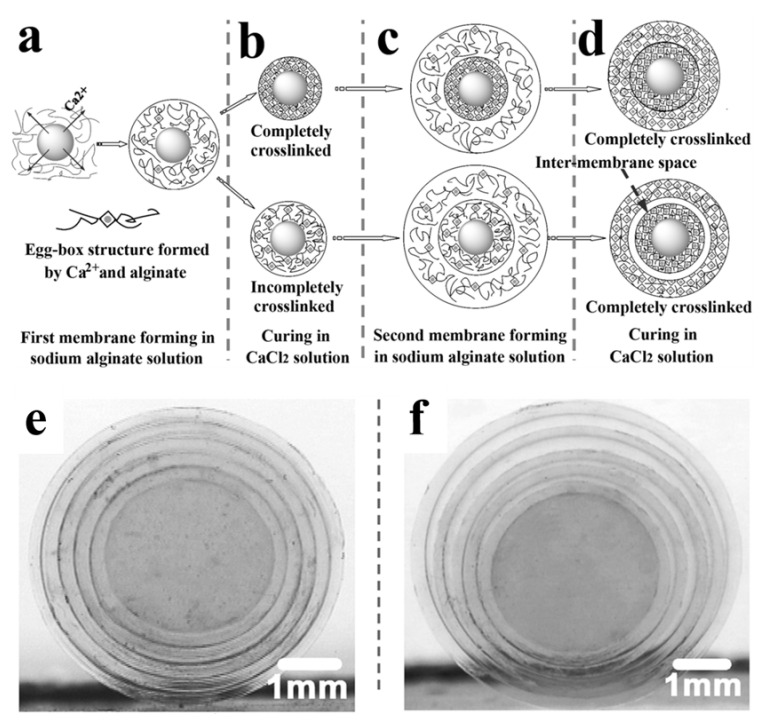
Schematic illustration of the preparation of alginate multilayer hydrogels. The partly crosslinked layer (membrane) formed with the egg-box structure in the sodium alginate solution (**a**). The completely or incompletely crosslinked hydrogels (**b**). The second layer formed at the periphery of either completely or incompletely crosslinked hydrogel (**c**). The finally obtained double-membrane hydrogel with or without inter-membrane space (**d**). Photographs of the multi-membrane alginate hydrogels without (**e**) and with (**f**) inter-layer space. (Reproduced with permission from [24]. Royal Society of Chemistry, 2009).

In addition to common crosslinking agents, such as organic dibasic acids and polyols, metal ions can also be used as special crosslinking agents to promote the gelation of polymer solutions. Moreover, the introduction of different metal ions has different effects on the hydrogel structure [25,26,27]. For example, the preparation process and mechanism of an alginate multilayer hydrogel from Xu’s group [25] is roughly shown in Figure 2. First, an egg-box structure gel core of sodium alginate crosslinked by Ca^2+^ was prepared by diffusing Ca^2+^ into the sodium alginate solution to crosslink alginate molecule chains. Second, the above gel core was immersed in the sodium alginate solution for a given time to prepare a hydrogel layer, which was further cured in the Ca^2+^ solution. Finally, alginate-based multilayer hydrogels with the desired layers could be prepared by repeating the above-mentioned process. In addition, carboxymethyl cellulose and other polyanions can be crosslinked by Ca^2+^, Al^3+^, and other polyvalent inorganic cations to form hydrogels [27]. This LBL assembly method can also be used to prepare carboxymethyl cellulose multilayer hydrogels using AlCl_3_ aqueous solution as a crosslinking agent, showing good versatility [24].

For multilayer hydrogels from the ion crosslinking method, complete or incomplete crosslinking is essential for the successful preparation of inter-layer spaces. Complete or incomplete membrane crosslinking can be readily controlled by adjusting the crosslinking time (Figure 2). A hydrogel with an inter-membrane space can be obtained by fully crosslinking incompletely crosslinked alginate hydrogel layers in CaCl_2_ solution (Figure 2d). Every ion-crosslinked layer is independent of other layers in the hydrogels. Therefore, these multilayer hydrogels produced using the ion crosslinking method are expected to be used in investigating the co-culture of multiple cells, drug delivery, and tissue engineering due to their unique structure.

#### 2.1.3. LBL by Electrostatic Interaction

**Figure 3:**
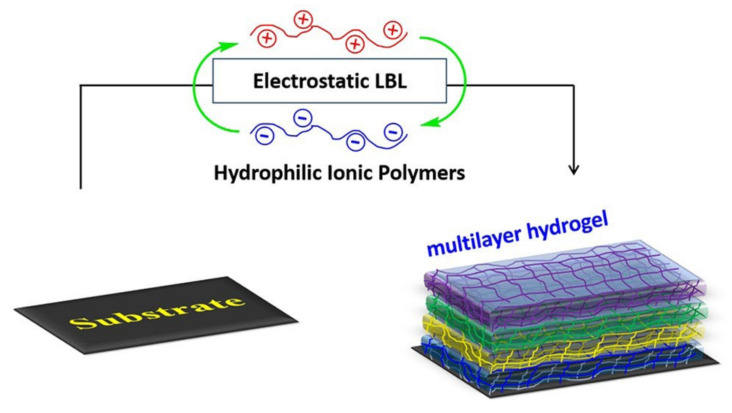
The scheme for formation of multilayer hydrogel from electrostatic LBL. (Reproduced with permission from [28]. John Wiley & Sons, 2020).

In addition to the introduction of crosslinking agents to prepare multilayer hydrogels, polyelectrolyte-based multilayer hydrogels can also be facilely prepared through simple electrostatic interactions [28,29]. Figure 3 shows the scheme for the formation of a multilayer hydrogel by electrostatic interaction. Positively and negatively charged polyelectrolytes can form hydrogel layers alternately on the substrate through electrostatic interaction. The key to electrostatic interaction is the mutual adsorption and surface charge reversal of positive and negative polyelectrolyte-based hydrogels. The concentration, pH, and temperature of polyelectrolytes are the most important factors affecting the formation and stability of multilayer hydrogels [28]. As the most commonly used LBL deposition strategy, electrostatic interaction has been widely studied and applied. Because electrostatic LBL assembly can be carried out in aqueous solutions, it is convenient to prepare LBL multilayers automatically using a LBL deposition machine [29]. These multilayer hydrogels produced using the electrostatic interaction method are widely used in different fields, including surface modification.

#### 2.1.4. LBL through Acid-Base Neutralization 

**Figure 4:**
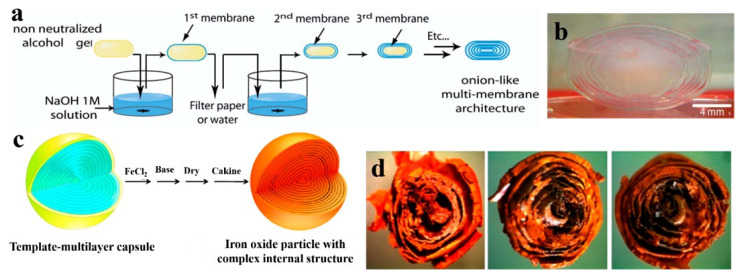
Process diagram of the preparation of chitosan multilayer hydrogel by LBL process through neutralization (**a**) and the photograph of the corresponding chitosan multilayer hydrogel (**b**). Schematic illustrating the iron oxide templating procedure (**c**) and photographs revealing the internal structure of iron oxide particles generated from the multilayer template) (**d**). (Reproduced with permission from [30,31]. Nature publishing group, 2008 and American Chemical Society, 2014).

Neutralization is an effective pathway to fabricate hydrogels, especially for acid-dissolved chitosan, by converting NH_3_^+^ in low pH solution to NH_2_ with the addition of a base solution. This can weaken the ionic repulsions between chitosan chains, resulting in physical cross-links through hydrogen bonding, hydrophobic interactions, and crystallite formation [30]. As early as in 2008, Alain Domard’s group reported a chitosan multilayer hydrogel using the interrupted neutralization process (Figure 4a). A chitosan physical alcohol gel was prepared by adding 1,2-propanediol aqueous solution to chitosan/HCl solution and the subsequent evaporation process. Then, NaOH aqueous solution was used to neutralize acid in the chitosan alcohol gel and form the first chitosan layer and inter-layer space for a given time. Finally, onion-like chitosan multilayer hydrogels with the desired layers (Figure 4b) were prepared by repeating the above-mentioned process (namely LBL).

In contrast to the above chitosan multilayer hydrogel, Shi’s group fabricated alginate/chitosan composite multilayer hydrogels via the interrupted neutralization of the as-prepared fluid-filled capsules with a polyelectrolyte shell layer [31]. First, a single drop of a chitosan solution was added to an alginate solution, which was further incubated to form a fluid-filled capsule with a chitosan/alginate layer through electrostatic attraction effect. Then, the capsule was neutralized with alkaline solution for some time to form a chitosan layer through the gelation of chitosan solution in the capsule. The corresponding multilayer hydrogels could be fabricated layer by layer through the repeating of the intermittent neutralization.

From the microscopic perspective, the semi-permeable polyelectrolyte complex shell layer from chitosan and alginate can retain chitosan solution inside the capsule, and the chitosan hydrogel layers cannot block the movement of OH^-^ in the alkaline aqueous solution to the inner chitosan solution. The OH^-^ group can convert NH_3_^+^ in the chitosan solution into NH_2_, resulting in the further formation of the chitosan hydrogel layer [32]. The quantity, thickness, and microstructure of multilayer hydrogels can be controlled by the concentration of alkali and contact time [30,31,32]. Interestingly, the prepared multilayer hydrogels can be used as templates to create hard particles with a complex internal structure, such as iron oxide particles (Figure 4c,d), and composite multilayer hydrogels consisting of organic-inorganic substances can be formed accordingly. Moreover, multilayer hydrogels prepared using the neutralization method have the advantages of uniform drug bearing, controllable inter-layer space, and good biocompatibility, showing potential application in the fields of cell culture and drug delivery.

**Figure 5:**
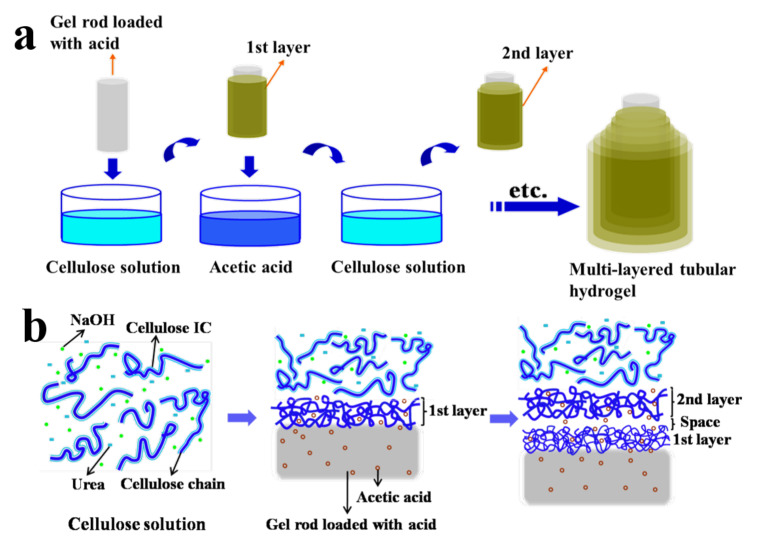
Preparation process of a cellulose multilayer hydrogel by a multi-step interrupted gelation (**a**) and the corresponding schematic model to describe the formation process (**b**). (Reproduced with permission from [34]. American Chemical Society, 2014).

As shown above, the general method to manufacture multilayer hydrogels from outside to inside was to utilize an interrupted chain condensation and contraction of an as-prepared hydrogel to form gel layers (namely LBL). However, this method is usually time-consuming and cannot readily load drugs [33]. To solve the above problems, the acid-base neutralization interaction can also be generalized to form hydrogel layers from the inside to the outside using gel-cores and other raw materials. As an example, the preparation process of a cellulose multilayer hydrogel from our previous work [34] is roughly shown in Figure 5. First, the as-prepared agarose gel rod was immersed into an acetic acid solution to load acetic acid as a coagulant. Subsequently, the gel rod loaded with acetic acid was immersed in a NaOH/urea dissolved cellulose solution to prepare the first cellulose layer. The gel rod with the first layer was immersed again in the acetic acid solution, so the new gel rod loaded with acetic acid again could be used to form the second cellulose layer. Finally, the cellulose multilayer hydrogels were fabricated by repeating this process (LBL).

Interestingly, a water-soluble inclusion complex (IC) associated with cellulose, NaOH, urea, and water occurs in the NaOH/urea solvent system at low temperature, which leads to cellulose dissolution [35]. When the cellulose IC was destroyed with acetic acid through the contact of acetic acid in the gel core and the cellulose solution, the strong inter-chain interactions of the exposed cellulose chains led to the rapid self-aggregation of cellulose and the formation of the first cellulose layer along the gel core (namely gelation or coagulation). Subsequently, the first cellulose layer was cured by re-immersion in acetic acid solution to load acetic acid, which could be used for the regeneration of the next cellulose hydrogel layer. Moreover, the inter-layer space was formed with the progress of the curing process. The fabrication process is facile and rapid, and the thickness and inter-layer spacing of the hydrogel can be controlled by adjusting the cellulose concentration, the diameter of the gel core, and the contact time. Multilayer cellulose hydrogels showed high compressive strength due to the dense packing of cellulose chains. The multilayer hydrogels prepared by the LBL process through acid-base neutralization have the advantages of stable gel structure, controllable shape, size, and thickness, and good biocompatibility, which are expected to be applied in cell culture and tissue engineering scaffolds [34].

**Figure 6:**
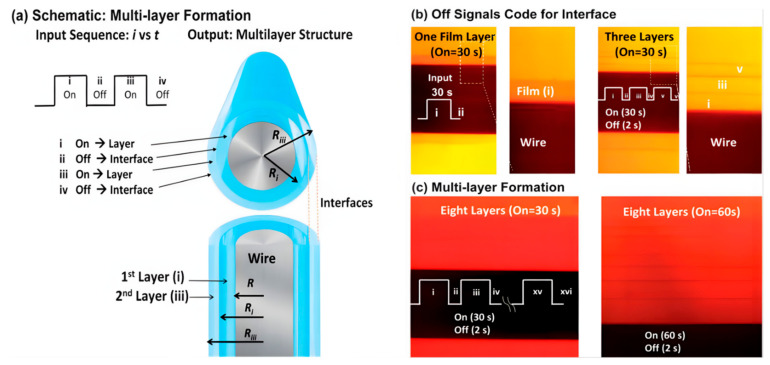
Chitosan multilayers generated by input sequences of “on-steps” (0.5 mA) and “off-steps” (0 mA). Schematic illustrating how electrical input controls the output multilayer structure (**a**). Off-steps (interruptions) code for interfaces (**b**). The left images of b show one layer and its enlarged photo on the wire by biasing a 30 s electrical input; the right images of b show a three-layered gel on a wire by three successive on–off sequences. The duration of on-steps controls the layer thickness (**c**). Images show eight layers with different thicknesses controlled by 30 s and 60 s on-steps, respectively. (Reproduced with permission from [36]. Royal Society of Chemistry, 2013).

Moreover, the electrochemical method can also be used to fabricate multilayer hydrogel through broadly defined acid-base neutralization interaction. Briefly, electrochemical synthesis is based on the use of electrochemical workstations to generate multilayer hydrogels through programming input electrical signals. The chitosan-based multilayer hydrogel from Shi’s group is used as an example [36] and its preparation process is shown in Figure 6a. First, a chitosan solution was prepared with the pH of 5 [37]. Secondly, a stainless-steel wire was adopted as the working electrode to immerse in the above chitosan solution, and a platinum wire was adopted as the counter electrode to connect the electrochemical workstation. Finally, chitosan multilayer hydrogels with different layers and thicknesses could be fabricated layer by layer using a pulsed electrical signal under the On–Off model.

This method enabled the assembly of the chitosan hydrogel in the cathode by a neutralization mechanism through the input of electrical signals [38,39]. Electrolysis can control the local pH [40], and the generation of OH^-^ at the cathode is believed to neutralize acidic chitosan solution and induce its localized sol–gel transition [41]. Physical crosslinks of the deposited chitosan hydrogels occurred in the crystalline regions [42]. Obviously, each interruption (off-step) generates an interface and a multilayer structure can be generated by an input sequence with multiple interrupts (Figure 6b). The duration of the on-step can control the thickness of the individual layers (Figure 6c). The above images demonstrate that the controllable multilayer hydrogels can be created using electronic input signals. Moreover, the simple inputs of the electrical signal do not change the solution compositions, making it more convenient to control and adjust [43]. Thus, this work provides an initial proof of principle that electronic codes and can be used to guide the assembly and control the hydrogel structure. Multilayer hydrogels prepared by this method provided new possibilities for tissue regeneration, multifunctional coating, and controlled drug delivery.

#### 2.1.5. LBL by Compound Methods

**Figure 7:**
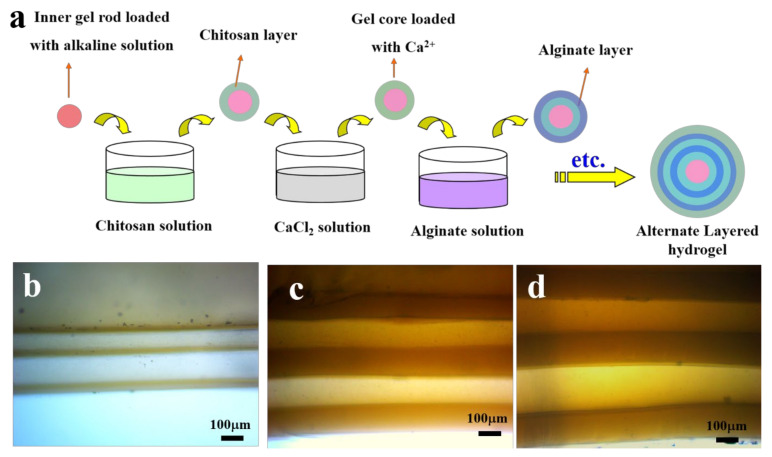
The preparation process of spherical alternate layered chitosan/alginate composite hydrogels (CACH) through acid-base neutralization and ion crosslinking (**a**), and photographs of the tubular CACH fabricated from different chitosan/alginate concentrations and soaking time (**b**–**d**). (Reproduced with permission from [45]. Elsevier, 2017).

It is well known that the blood vessel is a tri-layered substance with different components for each layer [44]. Layers from different raw materials are expected to have different properties for varied requirements; thus, mimicking blood-vessel like multilayer hydrogels is important and attractive. However, the fabrication of multilayer hydrogels with different layer components is difficult using a single method, as mentioned above, and the combination of several methods can be used during the LBL process to solve this problem. As an example, in one of our previous studies, alternate layered chitosan/alginate composite hydrogels (CACH) were fabricated successfully using the LBL process with the combination of acid-base neutralization for the formation of the chitosan layer and ion crosslinking for the alginate layer (Figure 7a) [45]. The CACH was constructed by repeating the alternate formation of chitosan and alginate gel layers. All the tubular CACH exhibited good appearance and controllable layers (Figure 7b–d). The layer thickness increased with the increase in chitosan or alginate concentrations and soaking time. Moreover, the CACH exhibited good architectural stability and biocompatibility towards endothelial cells, thus showing significant potential as a cell culture carrier and a matrix for the controlled release of molecules.

### 2.2. Non-LBL Methods for Multilayer Hydrogels

As mentioned above, LBL has been predominantly used to prepare multilayer hydrogels in recent years. Non-LBL methods (traditionally) have also been developed to prepare multilayer hydrogels. Two non-LBL assembling methods are described in the following.

#### 2.2.1. New Concept Welding

**Figure 8:**
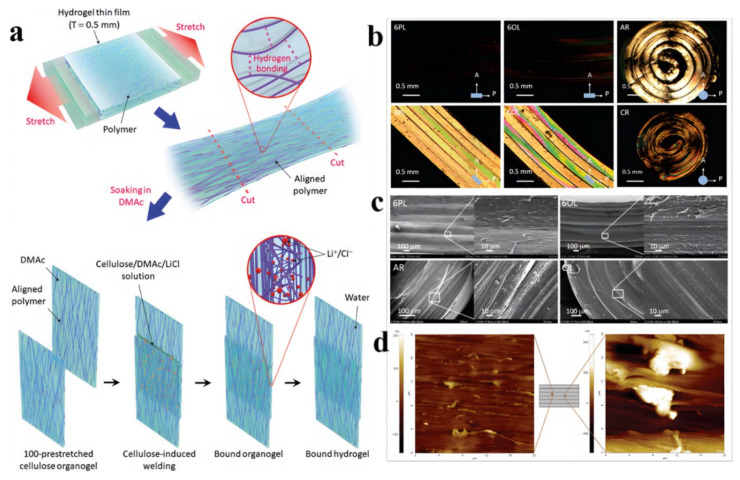
Design of weldable anisotropic cellulose multilayer hydrogels (**a**), POM images (taken in cross-polarized mode; A: analyzer; P: polarizer) (**b**) and SEM images (**c**) of the cross-section of 6PL, 6OL, AR, and CR multilayer hydrogels, and AFM images of the cross-section of 6PL hydrogel (**d**). (Reproduced with permission from [46]. Royal Society of Chemistry, 2019).

New concept welding is a method used to prepare anisotropic multilayer hydrogels by an ion-induced interfacial reconfiguration. Taking anisotropic cellulose multilayer hydrogels by Jeon’s group as an example [46], the design principle is shown in Figure 8a. First, cellulose was dissolved in a lithium chloride/N,N’-dimethylacetamide (LiCl/DMAc) mixed solution to obtain a cellulose solution, which was then cast on a mold to form an organogel layer through intermolecular hydrogen bonds (H-bonds). The resulting cellulose organogel was transformed into an isotropic cellulose hydrogel by immersion in water. The anisotropic cellulose hydrogel film was formed using axial force on the above isotropic hydrogel sheet, where the highly aligned polymer chains were fixed by H-bonds. Ion-induced welding by adding a LiCl/DMAc mixture was then used between the adjacent hydrogel layers through the realization of the intermolecular H-bond exchange at the interface. Finally, anisotropic cellulose multilayer hydrogels with different morphologies were prepared accordingly. Four different forms of anisotropic multilayer hydrogels (parallel laminated (PL), orthogonally laminated (OL), axially rolled (AR), and concentrically rolled (CR) multilayered hydrogels) were prepared through the hierarchical programming of cellulose chain orientation in hydrogels (Figure 8b,c), indicating the versatility of new concept welding.

In this method, a thin layer of cellulose/LiCl/DMAc solution was trapped at the interface of the two thin layers, and the interfacial LiCl gradually diffused into the hydrogel layer over time due to the concentration gradient, and reassembled the cellulose interface through the exchange of H-bonds. Highly aligned microfibers appeared in the bulk of a layer from 6PL gel, and the fibers in the interfacial regions of two layers were randomly distributed (Figure 8d). The hydrogel layers were completely integrated by an isotropic interfacial region with the possible occurrence of a full reconfiguration of the polymer chains. This facile method achieved the interface reconfiguration of hydrogels through ion-induced welding without an adverse effect on the highly aligned polymer orientation and the common adoption of covalent crosslinking, shedding light on the design of novel hydrogels used in the engineering and biomedical fields.

#### 2.2.2. Metal Ions Modulation

**Figure 9:**
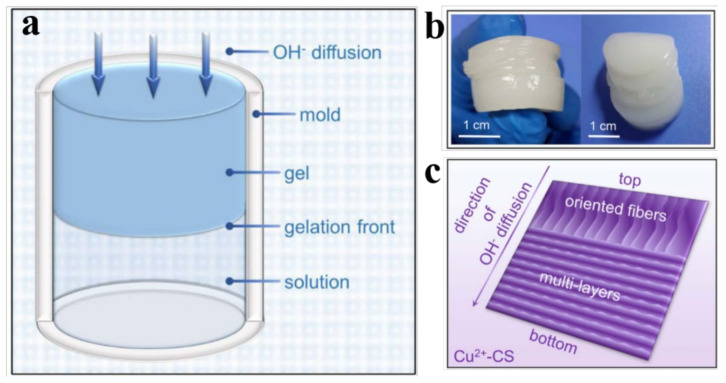
Schematic illustration of the formation of CS hydrogel (**a**), photograph of a CS multilayer hydrogel (**b**), and schematic illustration of the typical morphology of the copper-CS multilayer hydrogel (**c**). (Reproduced with permission from [47]. Nature publishing group, 2016).

Many polymers such as chitosan (CS) have functional groups including -NH_2_, which can coordinate with numerous metal ions through the chelation effect. Metal ions modulation can be used to prepare multilayer hydrogels through strong chelation interaction. A copper-chitosan composite multilayer hydrogel using Cu^2+^ modulation was fabricated by Wang’s group (Figure 9a) [47]. In this work, a Cu^2+^-CS solution was prepared first by adding CuCl_2_ powder to pure CS solution. Then, the above solution was filled in a single opening mold and immersed in an alkaline coagulation bath to complete the gelation process (Figure 9a). Finally, the resultant copper-CS multilayer hydrogel was formed and unloaded from the mold, which was repeatedly washed with deionized water to be neutral.

Pure CS could form a multilayer hydrogel through the addition of OH^−^ to the CS solution (Figure 9a,b) as mentioned above [48]. By comparison, the mechanism for copper-CS multilayer hydrogel formation with the structural transition differs and is summarized in the following. CS chain entanglement existed on the gel-sol interface (Figure 9c). Cu^2+^and CS can form a strong complex due to their strong affinity, resulting in the increased tendency of the volume of polymer zones to shrink. The gelation rate depends on the proximity of the gelation front to the system–coagulation interface. The introduction of Cu^2+^ ions increases the volume shrinkage of the CS bands, which causes a contraction at the gel–sol interface and enhances the disentanglement of macromolecules, resulting in the formation of a “clear space”. Two layers can be created with the gelation process by further diffusion of OH^−^ (Figure 9c). Thus, the copper-CS multilayer hydrogel can be fabricated accordingly, which had potential value in applications including copper-based fungicides, redox catalysts, and urea uptake.

## 3. Achievements and Practical Applications of Multilayer Hydrogels

Multilayer hydrogels with internal cavities and a complex internal structure have been widely applied in biomedical fields, including drug delivery, cell carrier or encapsulation, bacteria delivery, wound dressings (coatings), and bone repair. The achievements and practical applications of multilayer hydrogels have mainly focused on the biomedical field. Moreover, multilayer hydrogels have also been used in other fields, such as tuning ice nucleation and propagation, dye adsorptions, and forward-osmosis (FO) desalination, due to their unique structure, modifiable properties, and higher surface area.

### 3.1. Drug and Bioactive Substances Delivery 

Natural polymer-based multilayer hydrogels usually have excellent biocompatibility, and multilayer hydrogels in the forms of microspheres or capsules can effectively encapsulate and release drugs and bioactive substances. Inspired by biologic lipid bilayers [49], the development of multilayer hydrogels further deepened the study of drug delivery through the homogenization of the drug release in different layers, and by restricting the migration and diffusion of different drugs. The outer layer without drugs can effectively isolate the external environment and prevent the drug precipitating from the hydrogel surface, or the burst release of the drugs, which can prolong the drug release [50,51,52]. After arriving at the targeted sites, multilayer hydrogels can be induced to degrade by different stimuli-response pathways to achieve the precise drug release. Table 1 shows several kinds of multilayer hydrogels from different resources for the precise delivery and release of drugs by different pathways.

**Figure 10:**
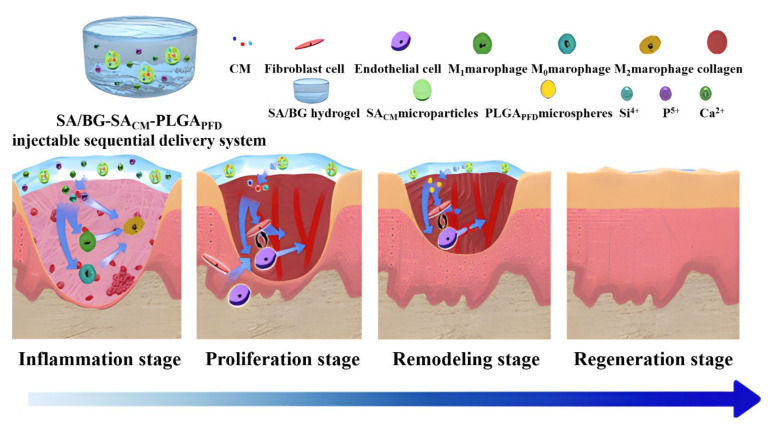
Scheme of a multilayer injectable hydrogel system that sequentially delivers bioactive substances for each wound-healing stage. (Reproduced with permission from [58]. American Chemical Society, 2020).

Wound healing is a dynamic and complex process that comprises several sequential phases, for which a number of drugs are effective. However, most of the current drug delivery systems were designed to treat only one phase of wound repair, ignoring the fact that every stage plays a critical role in the wound healing process. In an inspiring study, Maet al. reported that an injectable sodium alginate/bioglass (SA/BG) composite hydrogel can be used to carry SA microparticles containing a conditioned medium (CM) of cells (SA_CM_) [58]. Inside the SA_CM_ microparticles, poly(lactic-co-glycolic acid) (PLGA) microspheres containing pirfenidone (PFD) were encapsulated (PLGAPFD). This multilayer injectable hydrogel system (SA/BG-SACM-PLGA_PFD_) was designed to sequentially deliver bioactive molecules for satisfying the bioactivity requirement and timeline of each wound-healing stage (Figure 10).

### 3.2. Cell Encapsulation (Carrier or Bioreactors) and Bacteria Delivery

**Figure 11:**
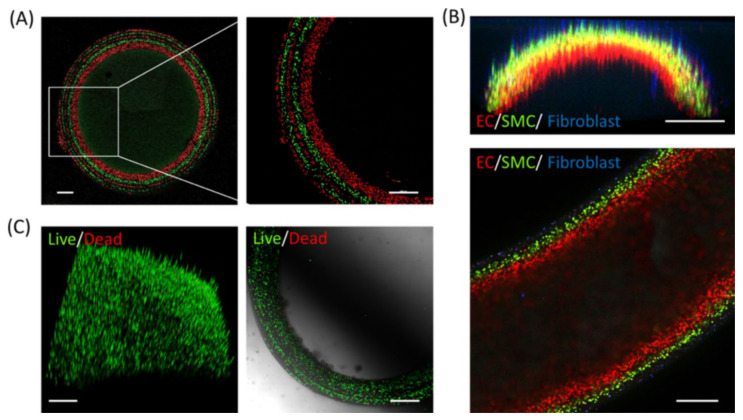
Cell encapsulation with multilayer hydrogels. (**A**) Images of a five-layer multilayer hydrogel tube embedded with fluorescence-tracked C2C12 cells of alternating color. (**B**) Cross-sectional (**top**) and longitudinal section (**bottom**) images of a three-layer tube embedded with HUVECs (red), SMCs (green), and fibroblasts (blue) in different layers. (**C**) 3D reconstruction (**left**) and cross-sectional (**right**) images of C2C12 cell-laden multilayer hydrogel walls stained for live (green) and dead (red) cells. Scale bar: (**A**–**C**) 500 μm. (Reproduced with permission from [60]. American Chemical Society, 2018).

The formation of multilayer hydrogels can sequentially and heterogeneously control the organization of cells, and the cavities between the layers can serve as good cell carriers or bioreactors [59]. The fast diffusion-induced gelation method was used by Sun’s group to fabricate multilayer hydrogels with controllable layer thickness for the encapsulation of viable cells [60]. Five layers of cells marked with alternate green/red fluorescence were assembled in a LBL fashion into a tubular structure by immersing the core gel into alternating solutions of each labeled cell (Figure 11A). The cells in each layer were separated by distinct boundaries, indicating limited mixing of the gel components at each step. Heterogeneous cell-laden multilayer hydrogel tubes were fabricated with HUVECs, SMCs, and fibroblasts, which were distributed from the inside to the outside of tubes to mimic native blood vessels (Figure 11B). Moreover, all the layers from the multilayer hydrogels exhibited high cell viability (>90%) according to the live–dead staining result (Figure 11C).

It is well known that the strong acid environment of the stomach is harmful to probiotics, and oral delivery of probiotics is a significant challenge. To address this challenge, Chen’s group prepared multilayer alginate hydrogel beads (MAHBs) by an emulsion method via ionic crosslinking between calcium ions and the carboxylic group of alginates, which can be used as an encapsulating material for oral delivery of a model probiotic bacterium *B. breve* [61]. MAHBs can be widely used as a carrier for probiotics oral delivery because they can significantly promote the viability of a variety of bacteria (including *B. breve*, *S. aureus*, and *E. coli*) at a low pH environment similar to that stomach, thus retaining the activity of the probiotics in the stomach. MAHBs can be utilized in the fermentation process, which is needed to release metabolite continuously and to avoid the burst release, and have been shown to be an excellent encapsulating material for oral administration.

### 3.3. Cartilage Repair

The need for bone repair materials has increased due to the complications associated with population aging. Hydrogels are often used as temporary fracture internal fixation materials due to their good mechanical properties, biocompatibility, and biodegradability [62]. Moreover, hydrogels are expected to treat cartilage diseases by mimicking the structural and functional characteristics of the natural extracellular matrix (ECM). It has been noted that multilayer hydrogels exhibit more advantages than ordinary single-layer hydrogels due to their unique structure, and layers with different and modifiable properties. Multilayer hydrogels can not only simulate the overall structure of cartilage, but also allow chondrocytes to migrate in the best form of tissue [63,64]. The internal structure of multilayer hydrogels can provide an appropriate microenvironment for the proliferation and differentiation of cells and microorganisms [65].

**Figure 12:**
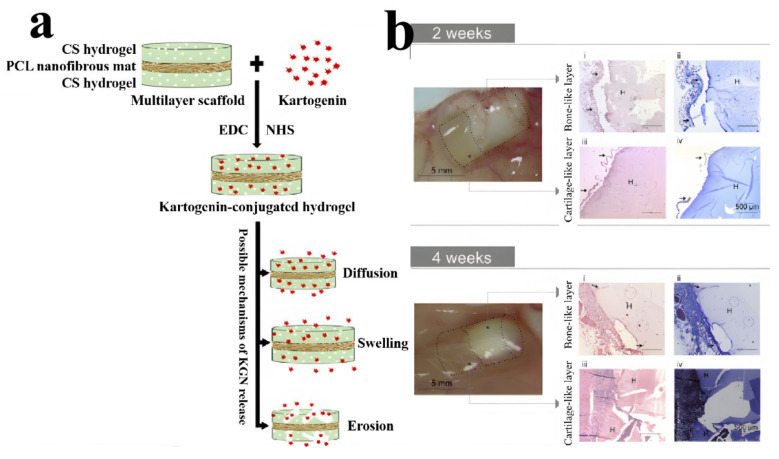
Schematic illustration of conjugation and mechanisms of KGN release from a MLS + K sample, showing three possible mechanisms (diffusion, swelling, and erosion or degradation) that are responsible for KGN release (**a**). Macroscopic images of the explants after implantation of (LAGG/LAGG-HAp 20%) hydrogels (H) in the dorsum of the mice for 2 weeks and 4 weeks (**b**). (Reproduced with permission from [66,67]. Elsevier, 2021, 2018).

Recently, Nasr-Esfahani’s group successfully constructed a chitosan/polycaprolactone multilayer hydrogel as a sustained Kartogenin (KGN) delivery system for cartilage engineering [66]. KGN was released from the hydrogels by three different mechanisms consisting of diffusion, swelling and erosion, or degradation (Figure 12a). KGN-conjugated multilayer materials (MLS + K) showed lower swelling ability and higher compressive modulus with gradual release of KGN in a longer retention time, which not only facilitated the effective treatment, but also provided a suitable mechanical structure for cartilage engineering and osteoarthritis treatment. Multilayer systems capable of simultaneous dual tissue formation were crucial for the regeneration of the osteochondral (OC) unit. Pereira et al. developed bi-layered hydrogel composites (BHCs) via the combination of two structurally stratified layers from nature-derived gellan-gum (GG) and hydroxyapatite (HAp) [67]. Either low acyl GG (LAGG) alone or in combination with high acyl GG (HAGG) were used for the fabrication of cartilage-like layers, and LAGG incorporating different ratios of HAp were used to prepare bone-like layers. GG-based layers and HAp reinforcement created a resilient bilayered structure with an interfacial region, which was not only able to integrate dissimilar zones, but also provided good stability during the degradation process. The BHC had good integration with surrounding tissues, and provided support for cartilage and bone-like tissue formation (Figure 12b), showing its feasibility as a osteochondral substitute with unique features for osteochondral regeneration.

### 3.4. Medical Dressings or Coatings

As mentioned above, wound healing remains a challenge in the biomedical field, which can be primarily be addressed by adopting appropriate wound care management. Wound dressings cannot only protect the wound from external damage, but also provide a suitable microenvironment for tissue regeneration. Hydrogels are suitable for the fabrication of medical dressing due to their excellent physical and chemical properties. Compared with monolayer or homogenous hydrogel wound dressings, multilayer hydrogel wound dressings can better promote wound healing because different layers can exhibit varied properties, which can satisfy different requirements of the top layer (barrier for bacterial transition and control of moist environment), middle layer (supply of controlled drug release for a long time and mass transfer limitations for drug molecules), and lower layer (absorption of the excess exudate, adhesion onto the wound surface, and support for new tissue formation) [68,69,70,71].

**Figure 13:**
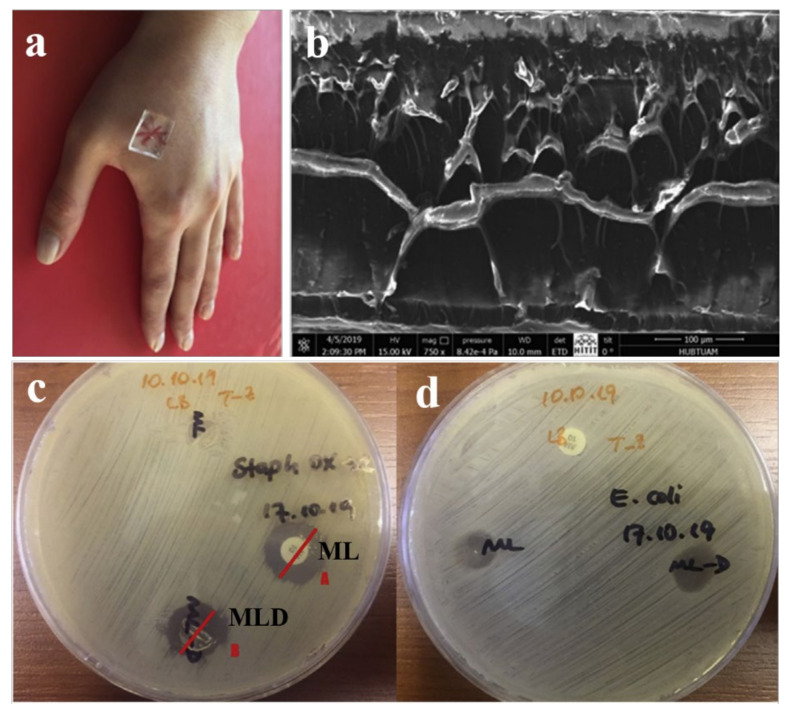
Photograph of a multilayer hydrogel as a wound healing dressing (**a**); the corresponding cross-sectional morphology (**b**); antimicrobial activities of ampicillin disc, ampicillin-loaded multilayer hydrogels (ML-D), and ML against oxacillin sensitive *S. aureus* (**c**); and antimicrobial activities of ampicillin disc, ML-D, and ML against *E. coli* (**d**). (Reproduced with permission from [72]. Elsevier, 2020).

Recently, Tamahkar and others fabricated a new type of four-layered hydrogel (ML) antibacterial wound dressing using carboxylated polyvinyl alcohol (PVA-C), gelatin (G), hyaluronic acid (HA), and G (Figure 13a,b) [72]. The PVA-C and G upper layers provided the most control and a physical barrier for microorganisms. The HA-based middle layer served as an antibiotic-loaded layer. The G lower layer was able to be used to release antibiotics and provide the removal of excess exudate from the wound site. ML hydrogels showed unique antibacterial performance against *S. aureus* and *E. coli* (Figure 13c,d). Moreover, the ML hydrogels showed antibacterial activity against oxacillin sensitivity, indicating that the novel wound dressings were an effective option for selective treatment of bacterial infections. Shokrollahi et al. prepared biocompatible electrospun multilayer nanofibrous dressings using PCL nanofibers as the first layer, hybrid nanofibers of chamomile/CECS/PVA and PCL as the second layer, and chamomile-loaded CECS/PVA as the third layer [73]. This multilayer dressing exhibited sufficient mechanical and swelling properties, and had excellent antibacterial efficiency due to the loading of chamomile, and could potentially be used for wound healing.

Türkŏglu et al. fabricated a wheat germ oil (WGO)-loaded multilayer hydrogel dressing by cross-linking sodium alginate (SA) with poly(ethylene glycol) diglycidyl ether (PEGDGE) on textile nonwovens [74]. This multilayer hydrogel showed rapid and positive swelling properties with an interconnected network of pores, and the resultant product was able to support the treatment of burns and wounds with medium to high exudate, and thus may be a promising alternative to conventional products in the wound healing field.

In addition to dressings, coatings from organic and inorganic materials can also be considered an excellent strategy to prevent bacterial adhesion, bacterial infection, and subsequent biofilm formation [75]. Multilayer hydrogels are able to extend the application of multifunctional biomedical coatings with a long use time due to their unique structure. Zhao et al. prepared an antibacterial and biocompatible multilayer biomedical coating by alternate deposition of chitosan (CS) and sodium carboxymethyl cellulose (CMC), which can be used to heal damage [76]. This multilayer coating exhibited high antibacterial properties by adsorbing the negative charge on the surface of bacteria, and fast and efficient self-healing properties through H-bonds and electrostatic attraction under specific stimuli. These features enabled the CS/CMC multilayer polyelectrolyte coating to have an extended lifespan, showing potential as a novel functional biomedical material.

**Figure 14:**
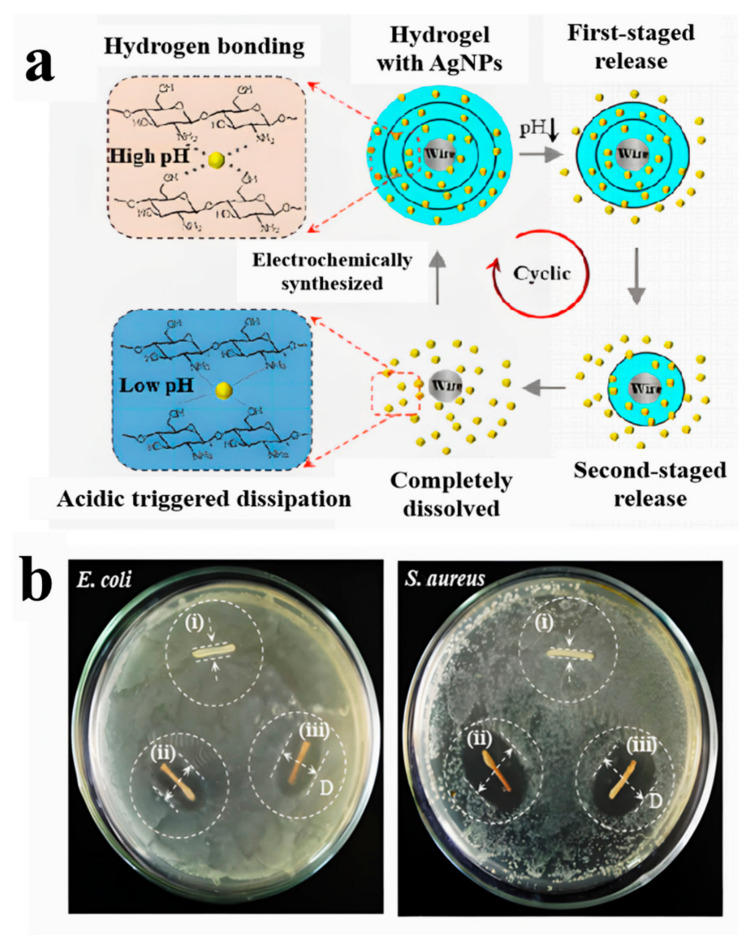
Schematic illustration of the staged release processes of AgNPs from a chitosan multilayer hydrogel based on acidic triggered dissolution of the hybrid coating layer by layer (**a**). Antibacterial activities of chitosan hydrogels with distinct compositions (**b**). i, bare 1-layer chitosan; ii, 1-layer chitosan loaded with AgNPs; iii, 3-layer chitosan loaded with AgNPs. (Reproduced with permission from [77]. Elsevier, 2021).

Shi’s group fabricated a chitosan/silver nanoparticle (AgNP) multilayer hydrogel coating via the combination of in situ synthesis of AgNPs on a pre-deposited chitosan multilayer hydrogel [77]. The coating conferred antibacterial properties by embedding AgNPs into the chitosan hydrogel network, using the ability of chitosan to adsorb and stabilize metal salts and sterilization by silver ion diffusion. The nanocomposite multilayer hydrogel coating exhibited a staged release behavior of AgNPs based on acidic triggered dissolution of chitosan hydrogel layer by layer due to its unique layered structure (Figure 14a). The obtained AgNPs with a narrow size of ~15 nm were evenly distributed throughout the hydrogel matrix to confer the multilayer hydrogel with excellent antibacterial properties (Figure 14b). This antibacterial multilayer hydrogel showed significant potential either to be used as a new coating material for the interfacial improvement of implants or as a wound dressing.

### 3.5. Other Fields

In addition to the large number of achievements and applications in the biomedical field, multilayer hydrogels can also be used in other fields. Three selected different application fields are introduced in the following.

**Figure 15:**
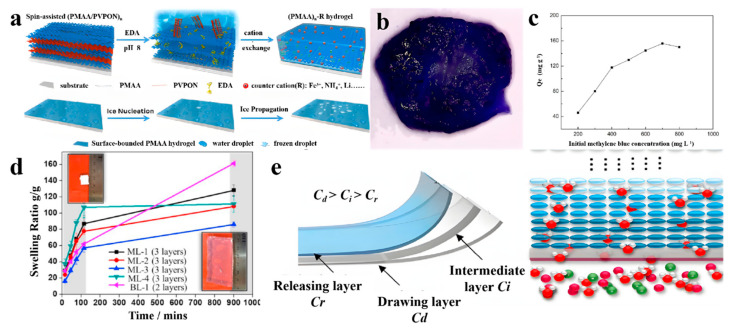
Schematic illustration of the fabrication of (PMAA)n-R multilayer hydrogels with different counterions (“R” denotes the type of counterion) and illustration of ice nucleation and ice propagation on (PMAA)n hydrogel surfaces (**a**); a photograph of the cross-section for the SAA2 multilayer hydrogel bead in methylene blue solution after adsorption of 72 h (**b**); and the corresponding effect of initial concentration of methylene blue on its adsorption capacity (**c**). Multilayer hydrogels with different configurations of layers along the direction of water transport; the inserted figures represent the ML-1 sample at t = 0 (top left) and t = 900 min (bottom right), respectively (**d**), and multilayer design with gradual reduction of SA concentration along the water transport pathway (**e**). (Reproduced with permission from [78,79,81]. American Chemical Society 2018, Tech Science Press 2019, Elsevier 2019).

The tuning of both ice nucleation and ice propagation via a simple anti-icing coating method is an important research topic, and was first investigated by Guo et al. using multilayer hydrogels [78]. Figure 15a shows the fabrication of poly(methacrylic acid) (PMAA)n multilayer hydrogels (n is the bilayer number). They first prepared a hydrogen-bonded multilayer of PMAA/poly(N-vinylpyrrolidone) (PVPON) at pH = 2.5 based on a LBL deposition approach. The neighboring PMAA layers were crosslinked with ethylenediamine (EDA), followed by the removal of the sacrificial template layers of PVPON at pH = 8.0. The ice nucleation and subsequent ice propagation on PMAA hydrogels with different counterions were investigated accordingly. The removal of dyes from effluents is also an important and urgent area of research, and hydrogels are important adsorption materials due to their advantages of low cost, high efficiency, and easy handling. Multilayer structures can increase the adsorption area for dyes such as methylene blue. In previous work, Chen et al. fabricated a novel multilayer composite hydrogel bead using alginate, acrylamide, and attapulgite for dye adsorption (Figure 15b) [79]. The multilayer hydrogels effectively adsorbed methylene blue and the maximum adsorption capacity reached 155.7 mg/g. (Figure 15c). These hydrogels are a promising adsorption material for dye-contaminated water treatment. Moreover, multilayer hydrogel capsules were also reported to load and release solutes including dyes via controlling temperature [80].

To solve the bottleneck of the lack of suitable draw agents in the development of FO desalination, Zeng et al. developed a multilayer temperature-responsive hydrogel on the basis of poly(N-isopropylacrylamide-co-sodium acrylate) (P(NIPAAm-co-SA)) [81]. The multilayer hydrogel was completely dry and white before the test (t = 0), which then swelled and became transparent (Figure 15d, inserts). The corresponding swelling curves were delineated into the initial fast swelling stage and the subsequent steady stages after 150 min (Figure 15d), indicating that the multilayer configuration did not affect the intrinsic swelling property of the P(NIPAAm-co-SA). The multilayer hydrogels showed a favorable performance for water storage with a reasonable mass transfer rate. The multilayer hydrogels consisted of a drawing layer with a high SA concentration for high osmotic pressure in the FO process, a releasing P-NIPAAm layer for fast water release, and intermediate layers for the reduction of the mass transfer resistance (Figure 15e). After dewatering and then cooling below the LCST, the P-NIPAAm releasing layer was expected to draw the water molecules from the intermediate layer more easily when compared with the bi-layer hydrogels. The multilayer hydrogel yielded a high capacity of water absorption and high permeable flux, which was very important for the development of hydrogel-based energy-efficient FO desalination.

## 4. Conclusions and Prospects

As a result of the continuous improvement in people’s living standards and the deepening of research in various fields, hydrogels have been widely developed and studied. Although many homogenous hydrogels with different excellent properties have been developed and improved continuously in recent decades, significant room remains for further development of hydrogels, because it is a challenge for these homogeneous hydrogels to simultaneously meet different needs due to the restrictions of their structure. Fortunately, novel multilayer hydrogels from different resources have emerged as required and become a new branch of hydrogels. Multilayer hydrogels have attracted significant attention and been studied and utilized in various fields due to their unique structure and excellent properties, as outlined above. Most preparation techniques (roughly classified as LBL and non-LBL) and related mechanisms of different multilayer hydrogels were cited and systematically discussed in this review. These impressive works will not only have a significant impact on the construction of multilayer hydrogels in the future, but also shed light on industrial processing for exploiting multilayer hydrogels in daily life.

However, in general, recent research and applications of multilayer hydrogels remain insufficient and lack maturity. Moreover, commercial products have not yet emerged because the existing methods for the production of multilayer hydrogels are relatively complex and difficult to apply in industrial settings. In addition, it is a challenge to implement the main biomedical applications, particularly in the clinical phase, because caution is needed in the evaluation of biomedical materials. Thus, further effort is required to develop novel methods and accelerate the evaluation of the long-term biocompatibility of multilayer hydrogels as implants. As a result of the continuous development of science and technology, prolific creativity, and effective cooperation, we believe that the preparation process of multilayer hydrogels will be optimized, and their properties will be continuously improved. Therefore, the issues relating to industrialization and biomedical applications of multilayer hydrogels may be solved based on the in-depth study of abundant resources, modifiable layers, and advanced technologies, and multilayer hydrogels can be eventually applied to every aspect of our lives.

## Figures and Tables

**Table 1:** Precise delivery and release of drugs by different multilayer hydrogels.

Drug Carriers	Drug Species	Release Pathways	Advantages	Reference
Poly(methacrylic acid)/poly(N-vinylpyrrolidone) multilayer hydrogel capsules	Nucleic acids	Ultrasound-triggered release	Higher effective loading capacity and controlled delivery of sensitive biomolecules	[53]
Poly(N-vinylcaprolactam) multilayer hydrogels	Sodium diclofenac	Increase in temperature	Multiple drug delivery	[54]
Chitosan multilayer hydrogel capsules	Doxorubicin	Adjustment of pH	Significant inhibition of the burst release and good biocompatibility,	[55]
Polycarboxymethyl-β-cyclodextrin (polyCM-β-CD)/polyetherimide (PEI) multilayer	Ofloxacin	Adjustment of pH	Controllable release in different media	[56]
Poly(methacrylic acid) (PMAA) multilayer hydrogel cubes	7-(benzylamino)-3,4-dihydro-pyrrolo[4,3,2-de]quinolin-8(1H)-one	Redox-triggered release	Long-term storage, combination of well-regulated drug release and shape-modulated drug delivery	[57]
